# Expulsion rate of intrauterine device: mediate vs. immediate puerperium period

**DOI:** 10.4274/jtgga.galenos.2020.2020.0037

**Published:** 2020-09-03

**Authors:** Juliano Terra Hochmuller, Karina Souza Lopes, Cristina Aparecida Falbo Guazzelli, Mariana Kefalas Oliveira Gomes, Edward Araujo Júnior, Alberto Borges Peixoto

**Affiliations:** 1 Mário Palmério University Hospital, University of Uberaba (UNIUBE), Uberaba-MG, Brazil; 2 Department of Obstetrics, Paulista School of Medicine - Federal University of São Paulo (EPM-UNIFESP), São Paulo-SP, Brazil; 3 Department of Obstetrics and Gynecology, Federal University of Triângulo Mineiro (UFTM), Uberaba-MG, Brazil; 4 Medical course, Municipal University of São Caetano do Sul (USCS), São Paulo-SP, Brazil

**Keywords:** Intrauterine device, contraception, puerperium, delivery, expulsion, postpartum

## Abstract

**Objective:**

To evaluate the rate of expulsion of intrauterine device (IUD) inserted during the immediate and mediate puerperium. To evaluate whether the type of delivery is a predictor of expulsion of the IUD when inserted in the puerperium period.

**Material and Methods:**

This was a prospective observational study. Patients whose IUD (TCU-380 copper) was placed during the puerperal period were divided in two groups according to the time of placement: immediate and mediate puerperium. The decision regarding the time of IUD insertion was made in a non-randomized manner. Analysis was performed using chi-square, Mann-Whitney U, and Spearman’s correlation tests and logistic regression analysis.

**Results:**

The total rate of IUD expulsions was 28.8% (49/170). There was no significant association between the occurrence of expulsion and the timing of IUD insertion (immediate vs mediate puerperium; 26.6% vs 34.78%, p=0.296). Among patients experiencing expulsion, 79.6% (39/49) underwent insertion after vaginal delivery and 20.4% (10/49) during cesarean section (CS). The type of delivery was a significant predictor for IUD expulsion (p<0.0001). Vaginal delivery was fourfold more likely to be associated with IUD expulsion inserted in the puerperal period than CS (odds ratio: 4.23, 95% confidence interval: 1.94-9.25). There was no significant correlation between the period between IUD insertion and the diagnosis of expulsion in regard to number of pregnancies (r=-0.160, p=0.271) or gestational age at delivery (r=-0.058, p=0.939).

**Conclusion:**

Vaginal delivery was the most prevalent type of delivery in patients who underwent IUD insertion during the immediate and mediate puerperium. The risk of IUD expulsion after vaginal delivery was greater than CS.

## Introduction

The postpartum period is marked by a transition for the woman and her family, during which the new mother experiences physical adjustments as she returns to her prepregnancy state and psychosocial changes as a result of the presence of a new family member (1). Currently, the World Health Organization recommends and emphasizes early medical follow-up during this period, with the goal of preventing and reducing neonatal and maternal morbidity in this stage of the female reproductive cycle (2,3). One concern during the postpartum period is the possible occurrence of a new pregnancy in a short period of time, which may not only cause maternal-fetal complications but may also have serious psychological, social, and economic repercussions.

Pregnancy and the postpartum period are appropriate times to discuss contraception, because there is an increased motivation for its use. This moment favors the patient-physician relationship and the evaluation of individual contraceptive needs. Physician guidance directly influences the woman’s decision about the use and type of contraception. Currently, some of the most commonly recommended options include long-term methods (e.g., long-acting reversible contraceptives), such as a copper or hormonal intrauterine device (IUD) and implant.

The copper IUD is one of the most widely used reversible contraceptive methods in the world, and it has an extremely low failure rate, with failure occurring in less than 1 in 100 women in the first year of use. It is indicated because of its ease of use, high efficiency, and association with safety and few side effects. One study showed that its use immediately after delivery was safe for both the mother and the newborn, with advantages including convenience and ease of insertion (3).

The literature indicates that the need for effective contraception in women immediately after delivery has been underestimated because, unfortunately, unplanned pregnancies can occur during this period. Recent research has shown that almost 50% of mothers return to having sex within six weeks of delivery, and many of these women do not use contraception (4).

Thus, there is a need for greater dissemination and understanding of the use of the IUD in the immediate postpartum period, as well as an assessment of its advantages and disadvantages. These questions encouraged us to analyze the use of this method in our practice.

## Material and Methods

This was a prospective observational study conducted in the Gynecology and Obstetrics Sector, from June 2018 to September 2019. The study was approved by the local research ethics committee (CAAE: 20502719.5. 0000.5145). Patients whose IUD were placed during puerperal period were divided in two groups according to the time of placement: immediate puerperium and mediate puerperium. A signed consent form was obtained from all patients.

The inclusion criteria included pregnant women ≥18 years old who expressed a desire to insert the IUD in the puerperal period. The exclusion criteria were the following: 1) IUD insertion between >72 hours and less than four weeks after delivery; 2) chorioamnionitis; 3) rupture of membranes >18 hours; 4) HIV; 5) puerperal sepsis; 6) puerperal endometritis; 7) abnormal uterine bleeding with uninvestigated etiology; 8) Mullerian anomaly; 9) postpartum hemorrhage; and 10) extensive laceration of the vagina.

The decision regarding the timing of IUD insertion (immediate puerperium period: between 10 and 15 minutes after placental delivery and 48 hours after delivery; mediate puerperium period: between 48 hours and six weeks after delivery) was made in a non-randomized manner. We used the TCU-380 copper IUD, which was inserted by medical residents in gynecology and obstetrics (first, second, and third years). We defined partial expulsion as the protrusion of the IUD from the external cervical os or visualization on transvaginal ultrasound of the distal end of the IUD below the internal os of the cervix (5).

### Insertion technique after vaginal delivery in anesthetized patients without the need for additional instruments

After uterine massage and prior to perineal repair, new sterile gloves were placed and oxytocin was administered [10 IU intravenously (iv)]. No specific antibiotic prophylaxis was used for the procedure. Subsequently, the IUD was removed from the insert. The IUD was placed between the index and middle fingers, and the opposite hand was inserted to stabilize the uterus externally. In the period between 10 and 15 minutes after placental delivery, the IUD was introduced until contact was made with the uterine fundus. To confirm that the IUD came into contact with the uterine fundus, the examiner used manual tactile perception through the placement of one hand on the uterine fundus. As the inner hand was being removed, a rotation of about 45 degrees clockwise or counterclockwise was performed, an act that was used to prevent the exteriorization of the IUD. Then, the threads were cut at the height of the external orifice of the cervix. The threads were visualized and trimmed on the follow-up visit four weeks after insertion. All cases in which the IUD wire was not visible inside the vaginal canal were referred for ultrasound examination to assess the position of the IUD. This technique was used in all patients who underwent vaginal delivery with peridural anesthesia.

### Insertion technique after vaginal delivery in non-anesthetized patients using Foerster or De Lee Forceps

This technique was used posteriorly and consisted of putting on sterile gloves, uterine massage, perineal repair, and administration of antibiotic prophylaxis and oxytocin (10 IU iv) as routine. Immediately after these steps, the IUD was removed from the insert. The IUD was then captured using De Lee’s Forceps, taking care not to activate the rack and to avoid damaging the copper, so that the sphere of the stem and the wires were parallel to the forceps. The upper tip of the IUD was placed flush with the tip end of the forceps. The wires were positioned away from the axis of the forceps, thus preventing them from becoming tangled or attached to the instrument when it was removed from the uterus. Then, using a hand or Doyan valve, the anterior lip of the cervix was exposed and visualized. A soft grip of the anterior lip of the cervix was performed using another De Lee’s Forceps. The cervix was pulled slightly, the IUD was inserted under direct visualization. Soon after, the hand that pulled the cervix was repositioned on the abdomen to stabilize the uterine fundus. Then, the IUD was precipitated to the uterine fundus, and the funicular position was confirmed with both the abdominal hand and the insertion hand. As the inner hand was being removed, a rotation of about 45 degrees clockwise or counterclockwise was performed, which was used to prevent the exteriorization of the IUD. The threads were cut at the height of the external orifice of the cervix and then visualized and trimmed during the follow-up visit four weeks after insertion. All cases in which the IUD wire was not visible inside the vaginal canal were referred for ultrasound examination to assess the position of the IUD.

### Insertion technique for cesarean delivery

After placental delivery, the IUD was inserted at the top of the uterine fundus either manually or using De Lee’s Forceps. Before hysterorrhaphy, the threads were incorporated in the lower segment of the uterus to allow them to hang naturally through the cervix during the puerperium period. Before hysterorrhaphy was performed, the IUD was confirmed as being retained in the fundus. The threads were visualized and trimmed during the follow-up visit four weeks after insertion. All cases in which the IUD wire was not visible inside the vaginal canal were referred for ultrasound examination to assess the position of the IUD.

The following variables were evaluated: age, number of pregnancies, number of deliveries, number of abortions, gestational age at delivery, type of delivery, time between IUD insertion and ultrasound examination, rate of IUD expulsion, time to diagnosis of IUD expulsion, rate of false path of IUD.

### Statistical analysis

The data were analyzed using SPSS, version 20.0 (IBM Inc., Chicago, IL, USA) and Prisma GraphPad version 7.0 (GraphPad Software, San Diego, CA, USA). The quantitative variables were initially subjected to the Kolmogorov-Smirnov normality test and were presented as median and the interquartile range; 25^th^ and 75^th^ percentiles. Categorical variables were described with absolute and percentage frequencies and represented in tables and graphs. To assess the difference between categorical variables and their proportions, a chi-square test was used. The Mann-Whitney U test was used to analyze continuous variables. To perform the correlation between continuous variables, Spearman’s correlation test was used. Logistic regression was performed to determine the best predictors for IUD misplacement. The level of significance (p) for all tests was <0.05.

## Results

**Figure 1:**
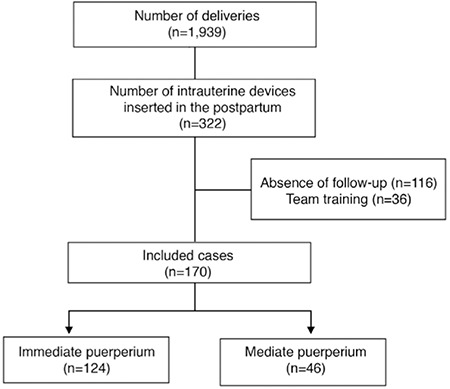
Study recruitment flowchart showing included and excluded cases

Between June 2018 and September 2019, 1,939 deliveries occurred in our service. During this period, 322 copper-T IUDs were inserted. A total of 152 cases were excluded because of lack of clinical follow-up after insertion. Thus, 170 cases were included in the final statistical analysis. The included cases were divided into two groups according to the IUD insertion period: insertion during the immediate puerperium period (n=124) and insertion during the mediate puerperium period (n=46; Figure 1).

There was no significant difference between IUD insertion timing groups (immediate vs mediate puerperium) and age (p=0.174), number of pregnancies (p=0.855), parity (p=0.896), number of abortions (p=0.570), gestational age at delivery (p=0.570), time between IUD insertion and ultrasound examination (p=0.179), and time between IUD insertion and the diagnosis of IUD expulsion (p=0.751; Table 1).

**Figure 2:**
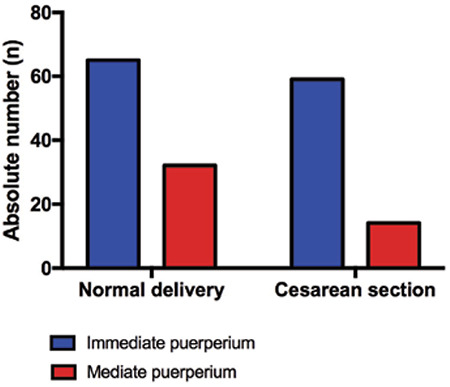
Bar graph showing the association between intrauterine device insertion and type of delivery. Chisquare, p<0.05

A significant association was observed between the IUD insertion and type of delivery (p=0.044). The rate of vaginal deliveries was higher in those undergoing immediate puerperium IUD insertion (52.42 vs 47.58%) and mediate puerperium IUD insertion (69.57 vs 30.43%) compared with the rate of cesarean sections (Figure 2).

In the whole cohort the overall rate of expulsion of IUDs was 28.8% (49/170). There was no significant association found between the occurrence of expulsion of the IUD and the timing of insertion (immediate puerperium vs mediate puerperium; 26.6% vs 34.78%, p=0.296), either clinically or with transvaginal ultrasound (Table 1). There was no significant association of IUD false path with either the immediate or mediate puerperium groups (1.6% vs 0%, respectively) (Table 1). There were no cases of endometritis in either group.

A significant association was observed between the type of delivery and IUD position (p=0.0002). Among patients diagnosed with IUD expulsion, 79.6% (39/49) underwent insertion after vaginal delivery, whereas 20.4% (10/49) had the IUD inserted during cesarean section. However, both for patients who underwent IUD insertion after vaginal delivery (59.80% vs 40.20%) and for those who underwent insertion during cesarean section (86.30% vs 13.70%), the rate of a correctly positioned IUD was higher than the expulsion rate (Table 2).

All IUDs were inserted by medical residents in gynecology and obstetrics, in the first (n=4), second (n=4), and third (n=4) years of residency, and none had previous experience inserting an IUD in the puerperal period. Physicians received only theoretical training prior to the start of the study. The first case of IUD insertion after vaginal delivery in the immediate puerperium period, the first case inserted after vaginal delivery in the mediate puerperium period, and the first case inserted after cesarean section by each physician were excluded. No significant association was found between the level of training of the medical residents, who had the same level of IUD placement training, and the rate of IUD expulsion (p=0.626; Table 3).

**Figure 3:**
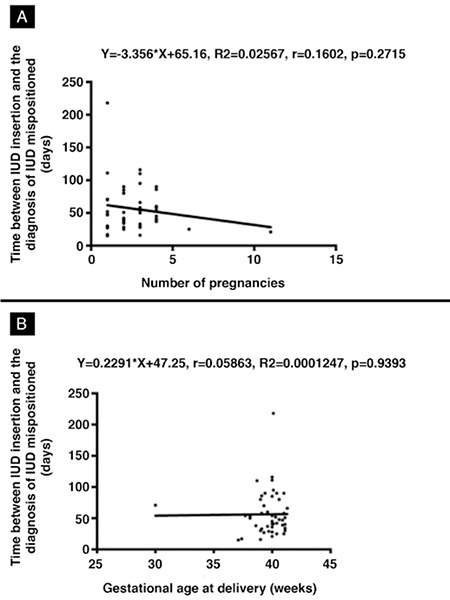
Scatter plot showing the correlation between the number of days between intrauterine device (IUD) insertion and the diagnosis of IUD expulsion in terms of number of pregnancies (A) and gestational age at delivery (B). Linear regression and Spearman’s correlation test, p<0.05

There was no significant correlation between the time between IUD insertion and the diagnosis of IUD expulsion and number of pregnancies (r=-0.160, p=0.271) or gestational age at delivery (r=-0.058, p=0.939; Figure 3).

A binary logistic regression was performed to determine whether the type of delivery in patients who had an IUD inserted in the puerperal period was a predictor of IUD expulsion. The model containing the type of delivery was significant for the prediction of IUD expulsion [χ^2^(1): 15.14, p<0.0001, R^2^ Nagelkerke: 0.12). Patients who delivered vaginally were 4.23 times more likely than patients who had cesarean sections to expel the IUD when it was inserted in the puerperal period [odds ratio (OR): 4.23, 95% confidence interval (CI): 1.94-9.25; Table 4).

A binary logistic regression analysis was also performed to determine whether the type of delivery in patients who had an IUD inserted in the immediate puerperium period was a predictor of expulsion of the IUD. The model containing the type of delivery was significant for the prediction of expulsion of the IUD when it was inserted in the immediate puerperium period [χ^2^(1): 20.58, p<0.0001, R^2^ Nagelkerke: 0.22). Patients who delivered vaginally were 8.17 times more likely than patients who had cesarean sections to expel the IUD when it was inserted in the immediate puerperium period (OR: 8.17, 95% CI: 2.89-23.11). In contrast, the model containing the type of delivery was not significant for the prediction of expulsion when the IUD was inserted in the mediate puerperium period [χ^2^(1): 0.008, p<0.930, R^2^ Nagelkerke: 0.000; Table 4).

## Discussion

This study evaluated if the type of delivery was a predictor of expulsion and rate of expulsion of IUD inserted during the immediate and mediate puerperium. The results have shown that the insertion of IUD during puerperal period is a safe technique, even when it is performed by medical professionals with no previous experience in puerperal IUD insertion. The copper-T IUD is classified as a long-acting, reversible contraceptive and is recommended by the American College of Obstetrics and Gynecology (ACOG) as one of the best contraceptive options in the immediate postpartum period (6). ACOG’s guideline on this method aims to improve the spacing of pregnancy, thus contributing to the improvement of maternal and child health care, especially in developing countries.

There was a low acceptance of IUD insertion in the mediate or immediate postpartum period in the population studied, with only 16.6% of women agreeing to its use. Another important finding was the loss of 47.2% of women who chose to insert an IUD after delivery and did not return for follow-up. Similar values have been reported previously with between 10% and 40% of puerperal women not returning for follow-up and that 40% to 70% of those who planned to use an IUD were unable to have it placed (7,8). These results show the benefit of offering this contraceptive method during the immediate postpartum period, as puerperal women have a lower likelihood of undergoing IUD insertion because of the difficulty of the procedure and lack of follow-up.

Our study found that IUD insertion in the immediate postpartum period was safe, which is in keepig with the findings of other studies (9,10). The advantages of this procedure include adequate retention of the IUD inside the uterus and the fact that is a safe and efficient, long-term contraceptive method. In our cohort, the rate of uterine perforation was low (1.6%). Conversely, previous studies have shown high rates of IUD translocation when the insertions were performed in the puerperal period (11,12). Moreover, there were no cases of endometritis diagnosed during the period of the present study.

One of the objectives was to evaluate the positioning of the IUD after its insertion, via ultrasound or physical examination, performed in the first 60 days. We found no difference in relation to age, number of pregnancies, or age among women of both groups. Interestingly, our findings showed that pregnant women who underwent vaginal delivery had a higher incidence of IUD acceptance than those who delivered via cesarean section.

One concern about IUD insertion after delivery is the higher rate of expulsion when compared with IUD insertion during other time periods. The rate of IUD expulsion was 28.8%, as determined by ultrasound or physical examination in the first 60 days. However, there was no difference in the frequency of IUD expulsion between insertions that took place during the mediate and immediate postpartum periods.

The definition of IUD expulsion is not always clear, and diagnostic criteria are rarely reported. Expulsions, as reported in the literature, may have been determined by a variety of methods, including clinical, physical, or ultrasound examinations. In a systematic review published in 2018, the authors reported that, depending on the time of IUD placement, there was a change in the incidence of expulsion. In the immediate, early, and interval of IUD insertion, the expulsion rate was 10.0%, 29.7%, and 1.9%, respectively, including all follow-up periods (13). The expulsion rate was 14.9% for vaginal deliveries, 3.6% higher than for cesarean deliveries, at all follow-up intervals (13). Similarly, in our study, patients who underwent IUD insertion after vaginal delivery were more likely to a have poorly positioned IUD than those who underwent cesarean section (OR: 8.17, 95% CI: 2.89-23.11).

Braaten et al. (14) reported that in approximately 10% of their users, the IUD was mispositioned. Currently, good positioning is considered when the end of the vertical IUD nail is above the internal os (15). In cases suspicious for poorly positioned IUD on ultrasound examination, symptoms such as pain and increased or irregular bleeding should trigger further investigation. There was no change in IUD expulsion rate when the IUD was inserted 4-6 weeks after delivery.

With regard to the type of delivery as a factor for IUD expulsion, in the present study 79.6% (39/49) of women underwent insertion after vaginal delivery, whereas 20.4% (10/49) had the IUD inserted during cesarean section, and in both types of delivery, the rate of adequate positioning was higher than the rate of poor positioning. A randomized comparative study, in which both immediate and late postpartum IUD insertions were performed, demonstrated that the failures were not influenced by the timing of insertion, cervical dilation, or distance between the apex of the IUD and the fundus of the uterine cavity (16).

In the present study women who underwent vaginal deliveries were 4.23 times more likely than women who underwent cesarean sections to expel the IUD when it was inserted in the puerperal period. Aoun et al. (17) reported the following risk factors for expulsion: history of previous expulsion of another copper-T IUD associated with a probability of a new expulsion of 30%, increased menstrual flow, and severe dysmenorrhea. Lopez et al. (18) in a systematic review compared immediate (within 10 minutes of placental delivery) versus early postpartum placement of the IUD and found no difference in expulsion rates. A trial from Uganda showed that expulsion was more likely in the immediate group, although the estimate was imprecise. In a meta-analysis, expulsion by six months post-delivery was more likely for the immediate group, but the confidence interval was wide (OR: 4.89, 95% CI: 1.47-16.32; participants: 210 in four separate studies). The authors concluded that the benefit of effective contraception immediately after delivery may outweigh the disadvantage of increased risk for expulsion.

O’Hanley and Huber (19) proposed that previous training was not necessary for IUD placement, which is in agreement with our study wherein the IUDs were placed by professionals with no previous experience in inserting puerperal IUD. Similarly, Cwiak and Cordes (20) reported that both experienced clinicians and interns can successfully insert IUDs within a medical residency program for gynecology and obstetrics. There was no significant association between the level of medical resident training in gynecology and obstetrics and the rate of expulsion of the puerperal IUD in the present study (p=0.626). Although earlier studies have shown that previous experience was not necessary for IUD insertion, in our opinion the overall high expulsion rates demonstrated in our study may have been in part due to the lack of prior training of the professionals. We did not evaluate the necessary experience in IUD insertion in the training period required to obtain lower expulsion rates. However, the low level of experience of the professionals in our study reflects the reality for many institutions sited in regions of low socio-economic status, where IUD insertion in the puerperal period can contribute to better family planning.

## Conclusion

In summary, vaginal delivery was the most prevalent type of delivery in patients who underwent IUD insertion during the immediate and mediate puerperium. The risk of IUD expulsion after vaginal delivery was greater than after cesarean section.

## Figures and Tables

**Table 1:**
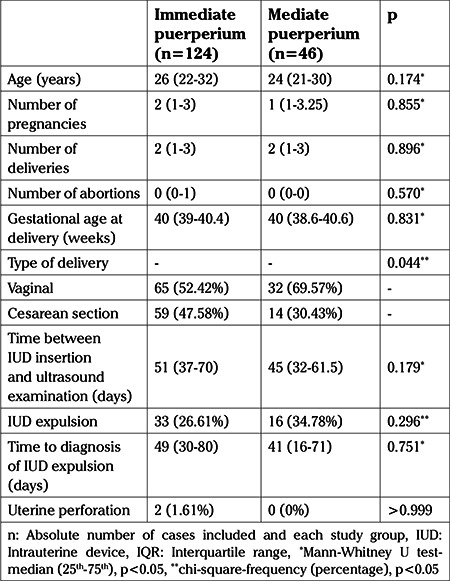
Clinical, obstetric, and intrauterine device characteristics of the studied population. Data are shown as median (interquartile range) or count and frequency (n, %)

**Table 2:**
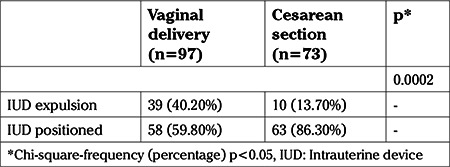
Association between type of delivery and intrauterine device positioning

**Table 3:**
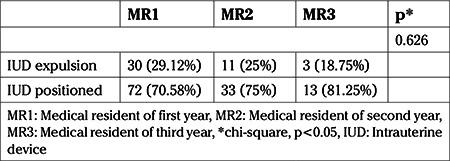
Association between the level of training of medical residents in gynecology and obstetrics and the rate of intrauterine device expulsion in the puerperal period

**Table 4:**
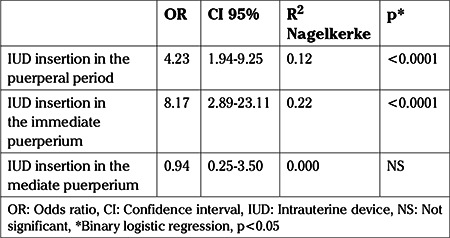
Odds ratio of intrauterine device expulsion in patients who underwent insertion in the puerperal period after vaginal delivery compared with cesarean delivery
